# Expression of ANGPTL4 in Nucleus Pulposus Tissues Is Associated with Intervertebral Disc Degeneration

**DOI:** 10.1155/2021/3532716

**Published:** 2021-11-28

**Authors:** Fan-jie Liu, Liang-yu Xie, Hua-zhong Li, Sheng-nan Cao, Yuan-zhen Chen, Bin-Shi, Dan-dan Wang

**Affiliations:** Bone Biomechanics Engineering Laboratory of Shandong Province, Shandong Medicinal Biotechnology Center (School of Biomedical Sciences), Neck-Shoulder and Lumbocrural Pain Hospital of Shandong First Medical University, Shandong First Medical University & Shandong Academy of Medical Sciences, Jinan, Shandong Province, China 250062

## Abstract

**Objective:**

Angiopoietin-like protein 4 (ANGPTL4), encoding a glycosylated secreted protein, has been reported to be closely related to many kinds of diseases, including diabetes, tumor, and some musculoskeletal pathologies, such as rheumatoid arthritis, osteoarthritis, and osteoporosis. The aim of the current study is to investigate the role of ANGPTL4 in intervertebral disc degeneration and analyze the association of ANGPTL4 expression with Pfirrmann grades.

**Methods:**

A total of 162 nucleus pulposus tissues were collected from lumbar intervertebral disc herniation patients undergoing interforaminal endoscopic surgery. Real-time quantitative PCR and western blot were performed to determine the mRNA and protein expression of ANGPTL4 in nucleus pulposus samples. Statistical analysis was performed to analyze the association of ANGPTL4 expression with Pfirrmann grades.

**Results:**

Based on the clinical data of 162 patients, results showed that Pfirrmann grades were significantly associated with patients' age (*r* = 0.162, *P* = 0.047) and were not significantly associated with patients' gender (*P* > 0.05). RT-qPCR and western blot results showed that the mRNA (*r* = 0.287, *P* < 0.05) and protein (*r* = 0.356, *P* < 0.05) expressions of ANGPTL4 were both closely associated with Pfirrmann grades. The expression of ANGPTL4 was remarkably increased in the groups of high IVDD Pfirrmann grades.

**Conclusion:**

The results demonstrated that ANGPTL4 expression was positively associated with the Pfirrmann grades and the severity of intervertebral disc degeneration. ANGPTL4 may be served as a candidate biomarker for intervertebral disc degeneration.

## 1. Introduction

Intervertebral disc degeneration (IVDD) is greatly common that has been regarded as a main cause of low back pain (LBP) in daily life. About 80% of adults are going to occur one episode of LBP [1], a prevalent, disabled disease generating serious health and socioeconomic burden for patients [2]. However, the pathogenesis and molecular mechanism that were associated with LBP have not been fully elucidated.

The gelatinous matrix of NP included collagen II and proteoglycans. The IVDD induces the loss of proteoglycan and tissue hydration. IVDD is characterized by chemokine production, extracellular matrix degradation, and cell phenotype changes [3]. Intervertebral disc degeneration was caused by synergistic effects of multiple factors, including genetic inheritance, nutritional delivery, and mechanical and inflammatory mediators [4]. Among these, the genetics play critical roles in the occurrence and progression of IVDD. Angiopoietin-like protein 4 (ANGPTL4) encodes a glycosylated, secreted protein containing a C-terminal fibrinogen domain, which is a number of angiopoietin-like protein (ANGPTL1-8) family [5]. It exists in many tissues including the liver, intestine, eye, and skeletal muscles [6]. ANGPTL4 as a regulator of multiple disease processes had been proved [7]. It has been reported that ANGPTL4 functions as a serum hormone that regulates glucose homeostasis, lipid metabolism, and insulin sensitivity [8]. It can also prevent tumor metastasis by inhibiting vascular growth and tumor cell invasion [9]. Recently, the reported studies showed that ANGPTL4 might be functional in some musculoskeletal pathologies, such as rheumatoid arthritis (RA), osteoarthritis, and osteoporosis [10]. Several scholars demonstrated the enhanced expression of ANGPTL4 in arthritis, particularly in bones and cartilage [11]. However, the relationship between ANGPTL4 and IVDD has not been discussed.

In the present study, we analyzed the ANGPTL4 expression level in IVDD tissues using RT-qPCR and western blot. Meanwhile, we also tested the relationship between ANGPTL4 expression level and Pfirrmann grades and evaluated its potential value for prevention and treatment of IVDD patients.

## 2. Material and Methods

### 2.1. Ethics Statement

This research was approved by the Ethics Committee of Shandong First Medical University & Shandong Academy of Medical Sciences. Written informed consent was obtained from each study participant.

### 2.2. Study Population

A total of 162 human NP tissues were obtained from IVDD patients who underwent interforaminal endoscopic surgery from Neck-Shoulder and Lumbocrural Pain Hospital of Shandong First Medical University and the 960th Hospital of PLA, China, from September 2020 to July 2021. The diagnosis of IVDD is based on clinical manifestations and related studies and is made by experienced experts. Inclusion criteria are as follows: (1) meet the clinical diagnostic criteria of IVDD and (2) the patient or the patient's family voluntarily provided a signed informed consent. Exclusion criteria for collected samples are as follows: (1) severe infectious diseases, (2) patients combined with tumors, and (3) autoimmune diseases and other systemic diseases. Patients were clarified as different degeneration grades via three experienced clinical experts by imaging examination materials. All patients were allowed to entry that had no tumor, tuberculosis, and other immune diseases.

### 2.3. Pfirrmann Grades of IVDD Patients

The diagnosis was confirmed by magnetic resonance imaging (MRI) [12]. The most universally used assessment of IVDD is the 5-grade classification system of IVDD suggested by Pfirrmann grades [13]. The degree of IVDD is accessed depending on the morphological structure and determined one to five grades (I-V) [14]. There are various aspects as clarified indexes, such as intervertebral disc structure, distinction of NP and AF, signal intensity, and height of intervertebral disc [15]. Based on Pfirrmann grades, all cases were determined with their degeneration grades and divided into different groups as follows: grade I: the shape of intervertebral disc keeps normal, no horizontal bands, and the distinction of nucleus and annulus is clear; grade II: the shape of disc is nonhomogeneous and horizontal bands, and the relationship of nucleus and annulus is vague; grade III: the shape is nonhomogeneous with blurring between nucleus and annulus, and annulus shape still can be recognizable; grade IV: nonhomogeneous shape with hypointensity, annulus shape not intact and distinction between nucleus and annulus impossible, and disc height usually decreased; and grade V: same as grade IV but with collapsed disc space [16]. The researchers verified that Pfirrmann grades II and III are mild because there is no loss of disc space height. Pfirrmann grade IV is regarded as moderate because of reducing intervertebral disc height and grade V is considered, and the disc had space collapse [17]. This criterion is a useful scoring tool to access IVDD.

### 2.4. mRNA Extraction and RT-qPCR

Total RNAs were extracted from degenerated NP tissues using TRIzol reagent (Takara, Dalian, China) according to the supplier's protocol. Using a NanoDrop spectrophotometer (ND-1000, Thermo Scientific, USA), total RNA concentration was evaluated by detecting absorbance at 260 mm. The first strand of cDNA was synthesized from total RNA using an OmniScript RT kit (Qiagen, Valencia, CA) according to the manufacturer's recommendations. The resulting cDNA was then subjected to real-time quantitative PCR for evaluation of the relative mRNA levels of ANGPTL4 and GAPDH. The primer sequences of ANGPTL4 were as follows: forward: 5′-GGCTCAGTGGACTTCAACCG-3′and reverse: 5′-CCGTGATGCTATGCACCTTCT-3′; GAPDH forward: 5′-CTCCTCCTGTTCGACAGTCAGC-3′and reverse: 5′-CCCAATACGACCAAATCCGTT-3′. 15 *μ*l PCR mix contained 0.5 *μ*l of cDNA from NP tissues, 7.5 *μ*l of 2× SYBR Green Master Mix (Invitrogen, Carlsbad, California, USA), and 200 nM of the appropriate oligonucleotide primers. Subsequently, gene-specific amplification was implemented by using an ABI 7900HT real-time PCR system (Life Technologies, Carlsbad, California, USA). PCR procedures are as follows: the mixture was preheated at 95°C for 10 min and then amplified at 95°C for 30 s, 60°C for 1 min, and 45 cycles. The resolution curve was measured at 95°C for 15 s, 60°C for 15 s, and 95°C for 15 s. The Ct value was called as the cycle number at which the fluorescence intensity arrives a certain threshold where amplification of each target gene was within the linear region of the reaction amplification curves [18]. Using the instrument's software (SDS 2.3), we obtained the Ct (threshold cycle) value of each NP sample. Relative expression level of ANGPTL4 was normalized by the Ct value of GAPDH (internal control). Using the comparative threshold cycle (2^−ΔCT^) method, the data were analyzed.

### 2.5. Western Blot

Human NP tissue was extracted from specimens. The number of 0.5 g tissue was placed into EP tubes. The protein was exacted by adding to 500 *μ*l of RIPA buffer (Thermo, Waltham, MA, USA) and 5 *μ*l PMSF (Beyotime) and protease inhibitor cocktail (Meilune). Centrifugation was performed at 12,000 rpm and kept for 20 min at 4°C. Using BCA test kit (Pierce Biotechnology), protein concentration will be measured. Next, in SDS-PAGE electrophoresis, 80 V electrophoresis was performed for 30 min, and then, the value was adjusted to 120 V electrophoresis for 60 min. Proteins were transferred to PVDF membranes (Bio-Rad) by electroblotting. The process was asked to regulate 300 mA for 100 min. Next, primary antibodies used are ANGPTL4 (1 : 500 dilution, Proteintech) from Abcam and GAPDH (1 : 2000 dilution, Proteintech), and 5% non-fat milk in TBST was disposed (50 mmol/l Tris, pH 7.6, 150 mmol/l Nacl, and 0.1% Tween-20) and then incubated in TBST with primary antibody overnight at 4°C. Subsequently, the membranes were incubated with corresponding secondary antibody (Cell Signaling Technology, Beverly, MA, USA; 1 : 2000) for 1 h. Protein band was detected by using Femto Maximum Sensitivity Substrate (Thermo Fisher Scientific) and Pierce ECL Western Blotting Substrate (Thermo Fisher Scientific).

### 2.6. Statistical Analysis

SPSS 25.0 software (IBM, Armonk, NY, USA) was used for statistical analysis. These data were expressed as the mean ± standard deviation. All experiments were performed in triplicate. Difference groups were compared by using one-way analysis of variance (ANOVA). All data used Spearman's correlation coefficient to assess the correlation between ANGPTL4 expression and Pfirrmann grades. A two-sided *P* value less than 0.05 was considered to be statistically significant.

## 3. Result

### 3.1. Clinical Data

The clinical data of 162 patients are shown in Table 1. Based on the analysis of Spearman's correlation coefficient, the results show that there is no statistical difference between the gender and Pfirrmann grades (*P* > 0.05). And the Pfirrmann grade is significantly associated with age (*P* = 0.047).

### 3.2. ANGPTL4 mRNA Expression Is Elevated in Degenerated NP

**Figure 1:**
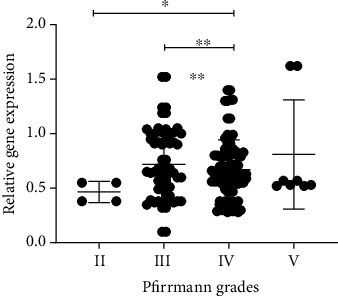
The mRNA expression of ANGPTL4 in NP tissues of different IVDD grades was evaluated by real-time quantitative PCR. The relative mRNA expression of ANGPTL4 is increased in the high IVDD grades, and its expression in the Pfirrmann IV group is significantly increased compared with Pfirrmann II and III; ^∗^*P* < 0.05 and ^∗∗^*P* < 0.01.

With the development of the Pfirrmann grades, ANGPTL4 expression is continually increasing (Figure 1). The statistical analysis shows that ANGPTL4 mRNA expression is significantly associated with Pfirrmann grades (*r* = 0.287; *P* < 0.05, Table 2).

### 3.3. ANGPTL4 Protein Expression Is Elevated in Degenerated NP

**Figure 2:**
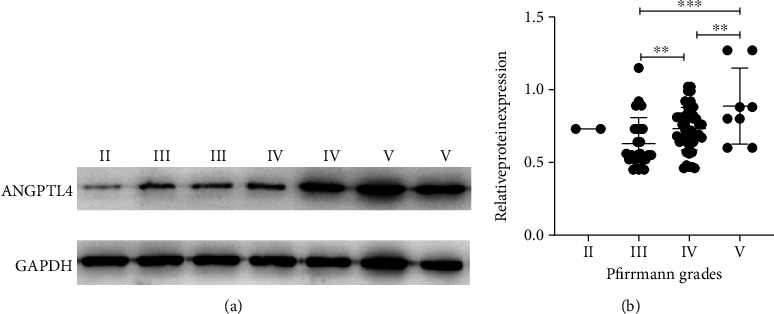
The protein expression of ANGPTL4 in NP tissues of different IVDD grades as assessed by western blot. (a) Representative result of ANGPTL4 protein expression in seven NP tissues. (b) The ANGPTL4 protein expression is remarkably increased in the groups of high IVDD grades. ^∗∗^*P* < 0.01 and ^∗∗∗^*P* < 0.001.

The western blot results show that the protein expression of ANGPTL4 in grade V is the highest and in grade II is the lowest (Figure 2(a)). The ANGPTL4 protein expression is gradually unregulated in groups of higher Pfirrmann grades (Figure 2(b)). The statistical analysis shows that ANGPTL4 protein expression is significantly associated with Pfirrmann grades (*r* = 0.365; *P* < 0.05, Table 3).

## 4. Discussion

IVDD is a chronic, high-incidence, and irreversible process [19]. IVDD is responsible for low back pain. Multiple scholars have researched the etiology of IVDD, which vital proteins and genes are functional and associated with this process. The mechanism of IVDD is complicated. As degenerative process occurs, intervertebral disc matrix proteoglycan could not prohibit vascular ingrowth. The vascularized granulation tissue allowed macrophages and mast cells to arrive the central part of intervertebral disc. The migrator promoted the expression of multiple cytokines, such as transforming growth factor *β*1 (TGF-*β*1), interleukin-1*β* (IL-1*β*), and tumor necrosis factor *α* (TNF-*α*) [20]. Based on the change of pathology, some studies found that IL-6, IL-2, and TNF-*α* were regarded as inflammatory biomarkers. In addition, P16, as an important regulator of cell growth and division, is regarded as a biomarker [21]. It is reported that microRNA might serve as potential biomarkers for the early diagnosis of IVDD [22]. Furthermore, some scholars found that serum CXCL12 could function as a biomarker of the early-mediate phase of IVDD development [23]. Progranulin (PGRN) also is determined as a target [22]. Hyaluronic acid, high mobility group box l (HMGB1), RPS4Y1, HSP90B1, and serum miR-155-5p are thought as biomarkers in the process of IVDD [24–28].

ANGPTL4 is a malfunctioned protein and potential treatment target in various diseases and has been found to be involved in several nonmetabolic and metabolic conditions, both physiological and pathological, including vascular permeability and angiogenesis, cell differentiation, lipid metabolism, tumor genesis, energy homeostasis, glucose homoeostasis, redox regulation, wound healing, and inflammation [29]. Although emerging studies have implicated that ANGPTL4 plays an essential role in tumor-associated activities [30], it is associated with multiple cancers, such as papillary thyroid cancer, breast cancer, and cutaneous melanoma [31]. In terms of the multifunction of ANGPTL4, some studies showed that ANGPTL4 may enhance the expression of MMP-1 and MMP-3 to decrease expression of type II collagen and aggrecan [32, 33]. Inflammation factors were induced by hypoxia. ANGPTL4 contribute to cartilage matrix degradation. Intervertebral disc is similar with articular chondrocytes in structure and function. Intervertebral disc is the largest avascular tissue in the whole body and existed in a lower oxygen tension than most tissues. Hypoxia-inducible factor (HIF) is a vital mediator of cellular responses to low oxygen tension. Hypoxic induction of ANGPTL4 by the HIF-1*α* isoform of HIF was initially described in cardiomyocytes, but also occurs in other musculoskeletal cells including monocytes, osteoclasts, and osteoblasts [10]. Hypoxia-induced factor-1*α* (HIF-1*α*) was abundantly expressed in NP [33]. In detail, we hypothesize that HIF-*α* in the degenerative NP tissues induces the expression ANGPTL4 to promote the secretion of inflammation factors and matrix metalloproteinase. The process may influence the extracellular matrix (ECM) to accelerate IVDD.

Meng et al. examined the expression of ANGPTL4, IGFBP1, IGFBP3, and IGFBP4 in patients with diabetic nephropathy. ANGPTL4 is considered as a biochemical marker for the detection of a diabetic kidney disease in patients with T2D [33]. Some papers show that IVDD has been correlated with T2D. Diabetic individuals are universal to have skeletal conditions, such as spinal stenosis, decreased disc height, and ossification of posterior longitudinal ligament [34, 35]. In view of the functional and wide-ranging characteristics of ANGPTL4, we performed the present study using a large number of samples of IVDD patients (162 cases) to analyze ANGPTL4 expression and its correlation with Pfirrmann grades. Results showed that the mRNA and protein expressions of ANGPTL4 were markedly increased in the groups of higher Pfirrmann grades. The expression of ANGPTL4 was significantly associated with Pfirrmann grades of IVDD patients. Our study suggested that ANGPTL4 may be a candidate biomarker involved in the pathogenesis of IVDD.

However, there are limitations in this study. Patients of Pfirrmann II grades usually choose conservative treatment, which limited the clinical sample collection. Patients of Pfirrmann V grades were seldomly diagnosed, because they choose operation treatment at the early stage. Besides, the function and mechanism of ANGPTL4 in the IVDD have not been elucidated, which needs further investigation.

## 5. Conclusion

In conclusion, our data demonstrated that the expression of mRNA and protein increases gradually with the increase of degeneration grade. ANGPTL4 may function as a candidate biomarker of the IVDD.

## Figures and Tables

**Table 1:** Clinical information of IVDD patients with different Pfirrmann grades.

Pfirrmann grades	*N*	Gender (male/female)	Age (mean ± SD)
II	4	2/2	19.00 ± 2.30
III	66	46/60	47.88 ± 12.92
IV	84	48/36	52.97 ± 12.74
V	8	4/4	41.50 ± 12.07
*r*		0.118	0.162
*P*		0.136	0.047^∗^

The correlation between age and IVDD grades was analyzed through Spearman's rank correlation. *n*: sample size; *r*: correlation coefficient; SD: standard deviation. ^∗^*P* < 0.05.

**Table 2:** The mRNA expression of ANGPTL4 in NP tissues of different IVDD grades.

Pfirrmann grades	II	III	IV	V	*r*	*P*
*n*	4	44	65	8		
ANGPTL4 mRNA expression (mean ± SD)	0.46 ± 0.09	0.58 ± 0.21	0.74 ± 0.24	0.81 ± 0.50	0.287	<0.001^∗∗∗^

*n*: sample size; *r*: correlation coefficient; SD: standard deviation. ^∗∗∗^*P* < 0.001.

**Table 3:** The protein expression of ANGPTL4 in NP tissues of different IVDD grades.

Pfirrmann grades	II	III	IV	V	*r*	*P*
*n*	2	25	46	8		
ANGPTL4 protein expression (mean ± SD)	0.73 ± 0.00	0.62 ± 0.17	0.73 ± 0.15	0.73 ± 0.15	0.365	<0.001^∗∗∗^

*n*: sample size; *r*: correlation coefficient; SD: standard deviation. ^∗∗∗^*P* < 0.001.

## Data Availability

The data used to support the findings of this study are available from the corresponding authors upon request.
